# Yearlong study of indoor VOC variability: insights into spatial, temporal, and contextual dynamics of indoor VOC exposure†

**DOI:** 10.1039/d4em00756e

**Published:** 2025-03-12

**Authors:** Thomas Warburton, Jacqueline F. Hamilton, Nicola Carslaw, Rosemary R. C. McEachan, Tiffany C. Yang, James R. Hopkins, Stephen J. Andrews, Alastair C. Lewis

**Affiliations:** a Wolfson Atmospheric Chemistry Laboratories, University of York UK tom.warburton@york.ac.uk; b National Centre for Atmospheric Science UK; c Department of Environment and Geography, University of York UK; d Bradford Institute for Health Research, Bradford Teaching, Hospitals NHS Foundation Trust UK

## Abstract

Volatile organic compounds (VOCs) are released from many sources indoors, with ingress of outdoor air being an additional source of these species indoors. We report indoor VOC concentrations for 124 homes in Bradford in the UK, collected between March 2023 and April 2024. Whole air samples were collected over 72 hours in the main living area of the home. Total VOC (TVOC) concentrations in the homes were highly variable, ranging from 100 μg m^−3^ to >8000 μg m^−3^ (median concentration ∼1000 μg m^−3^). Acetaldehyde and 1,3-butadiene concentrations in >75% of homes were found to be in exceedance of the 1 in 1 000 000 lifetime cancer risk threshold. Higher concentrations of benzene, toluene, ethylbenzene and xylene (BTEX) as well as trimethylbenzenes were found in urban houses (summed xylene median 2.35 μg m^−3^) compared to rural homes (summed xylene median 1.22 μg m^−3^, *p*-value = 0.02), driven by ingress of elevated outdoor BTEX and trimethylbenzenes (outdoor urban BTEX median 1.69 μg m^−3^, outdoor rural BTEX median 0.78 μg m^−3^). Inferred air change rate (ACR) exhibited a degree of seasonality, with average ACR varying between median values of 1.2 h^−1^ in the summer and 0.70 h^−1^ in winter. Time-averaged emission rate data provided additional insight compared to measured concentrations, such as seasonal variability, with highest total VOC time-averaged emission rates occurring in summer months (median 51 953 μg h^−1^), potentially a product of both increased occupancy times during school holidays as well as off-gassing of VOCs from surfaces. This comprehensive analysis underscores the critical role of seasonal, spatial, and contextual factors in shaping indoor VOC exposure, as well as potential health risks associated with consistently elevated concentrations of specific VOCs.

## Abstract 2

A comprehensive study of indoor VOCs, revealing how seasonality and location shape indoor VOC exposure.

## Graphical abstract

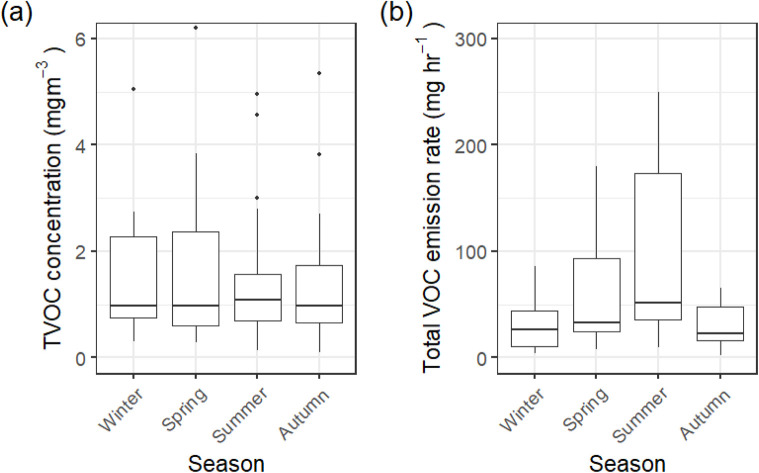

**Environmental significance**

This yearlong study provides critical insights into the environmental dynamics of indoor volatile organic compounds (VOCs), showcasing how urban–rural differences, seasonal patterns, and building characteristics influence indoor air composition. The findings emphasise the significance of understanding VOC sources and emission behaviours, not only for managing indoor environments but also for addressing their potential contributions to outdoor air pollution. Observations of VOC concentrations exceeding established benchmarks for lifetime cancer risk underscore the broader importance of reducing emissions from both indoor and outdoor sources to improve environmental quality.

## Introduction

Volatile organic compounds (VOC) are a class of air pollutants that are found both indoors and outdoors.^1–3^ Activities indoors such as cooking, using personal care products (PCPs), cleaning products and building and furnishing materials can all lead to VOC emissions; if these occur in poorly ventilated spaces then markedly elevated indoor concentrations can result.^4–7^ The same sources can also lead to the emission of semi-volatile organic compounds (SVOCs) which can undergo gas-to-particle phase partitioning, or produce new products following oxidation, as with VOCs. Both processes influence the total mass of particulate matter indoors, and hence the ability to cause harm to human health.^8,9^ As up to 90% of time is spent indoors,^10^ quantification of both VOC emissions and concentrations is an essential precursor to effective indoor air quality management.

Whilst acute VOC exposure has been associated with serious medical conditions, such as breathing difficulties and cardiac arrhythmia,^11,12^ the effects of long-term exposure to lower concentrations of VOCs remains relatively understudied. Ambient indoor concentrations of VOCs in typical UK settings are generally not high enough to give rise to acute health effects. Indoor measurements rarely show concentrations that exceed guidelines for acute effects, *e.g.* as in Shrubsole *et al.* (2019) and adopted by Public Health England as “UK guidelines for volatile organic compounds in indoor spaces”.^3,13–15^ However, as of yet, there are no long-term indoor data sets that allow for assessments of the health effects of exposure to VOCs with confidence.

Incidental release of VOCs through activities such as cooking and cleaning gives rise to sudden and often large increases in associated VOC concentrations. Concentrations decrease once the activity has concluded, dependent on variables such as room volume, air exchange rate, gas-surface partitioning of VOCs, and oxidation, and can take several hours.^6,16–19^ VOCs with the highest measured indoor concentrations in the UK are typically propane, butane and ethanol.^3,14^ A common source of propane and butane is from the use of aerosol products where they are propellants, including as home care and personal care products. Propane and butane concentrations greater than 3000 μg m^−3^ have previously been reported in homes.^3,14^ Ethanol is used as an ingredient in some personal care products such as hairspray, but also arises from fragrance and disinfectant use, as well as from cooking.^20–24^ Increasing ventilation rates and controlling source emissions can aid in mitigating high VOC concentrations indoors.^25,26^

Air change rate (ACR) is defined as the number of air changes within a volume over a time period, usually per hour. ACR is inherently difficult to accurately determine in residential settings, and is usually calculated/inferred through tracer or decay methods and models.^27,28^ Low ACRs have been shown to give rise to an increase in indoor VOC concentrations and hence exposure.^14,29,30^ Residential ACRs are typically around 0.5 h^−1^ to 2 h^−1^, however, ACRs in general can vary greatly depending on whether the space is in a residential or commercial setting, the time of year, the leakiness of the building envelope, and human behaviour, among other factors.^31–33^

Ambient temperature can impact indoor VOC exposure through increased material off-gassing of surface-bound VOCs.^34,35^ This process is uneven however,^36^ with newer building materials having generally higher off-gassing VOC emission rates than older materials regardless of temperature effects.

Indoor–outdoor (IO) ratios of VOCs can be used to highlight those VOCs which have large indoor concentrations; reactive species such as monoterpenes and other double-bond-containing hydrocarbons often have higher indoor concentrations compared to outdoors.^3,13,37,38^ These compounds are noteworthy because of the potential for secondary product formation when they are oxidised, such as secondary organic aerosols (SOAs).^39–41^ Chronic exposure to SOAs is potentially linked to an increase in mortality.^42,43^

Datasets on indoor VOC concentrations in UK homes remain sparse, particularly for lower-income households. Limited measurements have been conducted across the broader range of VOCs present in the UK.^3,13,14^ This paper reports on VOC concentrations observed in 124 occupied homes as part of the INGENIOUS (UnderstandING the sourcEs, traNsformations and fates of IndOor air pollUtantS) project.^44^ It quantifies the concentrations and time-averaged emission rates of indoor VOCs, and how these are influenced by seasonal effects of ACR.

## 2. Experimental and methodology

### 2.1 Study area

Bradford is a city in the West Yorkshire Combined Authority in the North of England, in the UK. With a population of 560 000,^45^ Bradford encompasses a large geographic envelope, with rural and urban areas often within short distances. Bradford is located east of the Pennine hills, and the city centre sits in a bowl-like position, flanked by inclines on almost all sides. A Clean Air Zone (CAZ) was introduced to Bradford within the outer ring road and extending up to Shipley in North Bradford in late-September 2022, currently the largest of its type in England outside of Greater London, covering 9.35 square miles. The extent of the CAZ, as well as a topographical presentation of the elevations surrounding Bradford are given in ESI Fig. S1.† The Bradford CAZ applies to all vehicles other than private cars and motorbikes in an effort to reduce vehicle-related emissions within the Bradford area, such as NO_*x*_. While it may be too soon to definitively identify the effects on outdoor air pollution of the introduction of the Bradford CAZ, preliminary results show a potential reduction in vehicle emissions since the introduction of the Bradford CAZ.^46^ More established CAZs, such as the Greater London Low Emission Zone (LEZ) and the Ultra-low Emission Zone (ULEZ), have been shown to reduce vehicle-related pollution levels more so than nearby CAZ-free areas.^47,48^ Given the potential for outdoor air penetration indoors, this could have a potential impact on indoor air quality in CAZ-enveloped areas.

Bradford's history is mainly industrial, being a centre of wool-based production and trade in the 1800s.^49^ Following the collapse of industrialisation in the UK, Bradford saw a decline in living standards, along with several other Northern English and other post-industrial cities across Europe.^50–52^ In the 21st century, Bradford has higher-than-average levels of socioeconomic disadvantage, as well as disproportionately lower life expectancy and health prospects when compared to the rest of England.^53–56^ Bradford is a highly diverse city with high levels of ethnic diversity.^57^

Studies have previously linked areas with higher deprivation levels with lower outdoor air quality across the entire UK and within countries and regions which make up the UK, especially so in comparable post-industrial cities.^58–61^ Outdoor air pollution in Bradford has been monitored continuously since 1999, with one continuous automatically-reporting monitoring site located on Mayo Avenue, Bradford measuring NO and NO_2_, as well as wind direction, speed and ambient temperature, forming part of the Defra national monitoring network.^62^ Despite over 20 years of continuous outdoor air pollution data collection, there are no reported datasets on indoor VOC concentrations for Bradford, or similar cities with high degrees of socioeconomic and ethnic diversity.

### 2.2 Participant selection and questionnaires

The methodology for participant selection and sampling regime, more broadly including the development and analysis of questionnaires, are detailed in Ikeda *et al.* (2023).^63^ In brief, 310 homes were monitored for indoor air pollutants using low-cost sensors.^44^ A subset of sampled homes participated in VOC analysis where a whole air canister was deployed and a time-integrated sample (up to 72 hours) was collected. The participants in this study were part of the Born in Bradford (BiB) birth cohort study.^64^ Homeowners were asked to complete home, health and behaviour surveys during sampling. This captured a large quantity of information that could be linked with each air sample. From these questionnaires, daily statistics of aerosol product and fragrance product use was taken for analysis in this study and summed for a total product use over the canister sampling period. A building audit was completed by BiB research assistants, capturing information about the different microenvironments in which samples were taken, of which the room volume was of specific interest to this study for the calculation of ACR (explained in Section 2.5). The full questionnaires are available in the ESI in Ikeda *et al.* (2023).^63^ Of the targeted 150 homes, a total of 124 homes had usable whole air samples taken for VOC analysis. All the samples had home, health, behaviour and building survey responses available. However, response rates to individual questions within the surveys were sometimes <100% (min 41%, max 100%, median 90%).

### 2.3 Sample preparation, collection and preparation for analysis

Whole air samples inside each home were collected using 6 L vacuum-intake stainless steel canisters treated internally with a proprietary silica-based ceramic (Entech, CA, USA). Flow-restrictive inlets (Entech, CA, USA) were used to time-integrate samples up to 72 hours (sampling flow rates through low surface area sapphire orifice restrictors varied between 1.4 and 1.9 mL min^−1^). Canisters were evacuated to 0.01 Pa (29.9 “Hg” vacuum gauge, 99.99% vacuum) prior to use. Canister valves were assessed for seal integrity using a vacuum gauge fitted to the sealed canister valve.

Canisters and flow-restrictive inlets, which remained paired throughout the study, were deployed in homes across Bradford from March 2023 to April 2024, with each sample collected over a 72 hour period. Canisters were consistently placed in the main living area, which was not always a designated ‘living room’ and often included open-plan spaces such as combined living, kitchen, and dining areas. Consequently, some canisters may have been exposed to episodic VOC emissions from activities such as cooking and food preparation. Canisters were positioned within the living area, no higher than 1 m above ground level and, where feasible, away from doorways. After sampling, the canisters were returned to the University of York for analysis.

### 2.4 Sample analysis

Samples were analysed following the method detailed in Warburton *et al.* (2023).^14^ Briefly, canister samples were initially diluted to 1 bar (gauge) with 6 L of humidified highly purified air free of VOCs (hereafter ‘blank gas’), produced by flowing compressed ambient air through a bed of platinum beads at >375 °C. 500 mL of diluted canister air was then drawn through a 16-port solenoid-actuated pneumatic valve manifold (Swagelok Company, OH, USA) at 15 mL min^−1^ into a custom-built thermal desorption unit (TDU), sequentially comprising a water trap, pre-concentration trap and finally a pre-injection focus trap. The water trap was held at −40 °C during sample intake, while the pre-concentration and focus traps were held at the lowest achievable temperature, always below −110 °C.

Following flash heating from the focus trap, dried, pre-concentrated and focused samples were injected into a two-column gas chromatograph (GC, Agilent 7890A, Agilent Technologies, CA, USA) fitted with flame ionisation detectors (FIDs) and a quadrupole mass spectrometer (QMS, Agilent 5977A, Agilent Technologies, CA, USA). Samples were first separated on a 60 m, 150 μm internal diameter (ID) VF-WAX column with a film thickness of 0.50 μm (Agilent Technologies, CA, USA) at 1.6 mL min^−1^ (helium carrier gas pressure of 35 psi). This resulted in a long elution of unresolved C_2_ to C_6_ hydrocarbons from the VF-WAX column, which were passed through a Deans switch (Agilent Technologies, CA, USA) to a 50 m, 320 μm ID Na_2_SO_4_-deactivated Al_2_O_3_ porous-layer open tubular (PLOT) column with a wall coating thickness of 5 μm (Agilent Technologies, CA, USA). Eluent from the PLOT column directly flowed through to an FID. Once the unresolved species had finished eluting through the VF-WAX column (at 8.3 minutes) the Deans switch diverted the analyte flow through a section of fused silica (2 m × 150 μm ID) to both balance column flows at the Deans switch and split analyte flow between a second FID and the QMS for simultaneous detection, through sections of 150 μm ID fused silica of length 0.91 m and 2.1 m, respectively.

A thirty-component mix of non-methane hydrocarbons (NMHCs) in nitrogen was used for sample calibration. Each VOC was at a mixing ratio of approximately 4 ppb, provided by the National Physical Laboratory, Teddington, UK (cylinder number D933515, hereafter referred to as ‘NPL 30’). Sampled VOCs contained within the NPL 30 mix were directly calibrated, with remaining VOCs calibrated using equivalent carbon responses. Table S1 (ESI†) gives the identify of the thirty directly-calibrated species. Following each canister batch, blank gas was sampled three times, followed by five NPL 30 calibrations and finally three carrier gas/internal samples (‘no flow blanks’). A no flow blank method involved the TDU operating with the sample volume set to 0 mL and the sample time set to 33.3 minutes, the time a regular 500 mL sample would take to be drawn. This process resulted in only carrier gas flowing through the TDU traps over the sampling period, resulting in canister sample correction for both blank gas diluent impurities (none found) and wider carrier gas and system impurities (consistently 0.95 μg m^−3^ benzene only). Following analysis, canisters were re-evacuated according to the previous method. Evacuated canisters were spot checked for impurities by filling from evacuated to 1 bar (gauge) with humidified blank gas and run on a regular canister sampling method.

Following GC analysis, a subset of *n* = 90 samples was further processed for greenhouse gas analysis, by flowing the canister samples at 600 mL min^−1^ into a laser absorption spectrometer (Ultraportable Greenhouse Gas Analyser, Los Gatos Research Inc., CA, USA). This additional analysis allowed carbon dioxide mixing ratios to be quantified.

Chromatograms for each sample were initially qualitatively analysed using MassHunter (Agilent Technologies, CA, USA) to assess the quality of chromatographic separation and resolution. Chromatograms were then integrated using *GCWerks* (GC Soft Inc., CA, USA). FID data was mostly used for peak integration and concentration data analysis. However, QMS data was required to deconvolve benzene, monoterpenes, chlorinated species and cyclosiloxanes. Over 120 VOC species were identified and included in the analysis. Instrument limits of detection (LOD) and limits of quantification (LOQ) were calculated using a signal-to-noise ratio of 3 : 1 and 10 : 1 respectively, and are shown in Table S2 (ESI†) for all VOCs measured by the instrument (including some otherwise not explicitly mentioned in this paper).

The entire VOC dataset is open-access and available at https://doi.org/10.15124/24fd1762-0e98-4773-a74c-7dd87ef59aa8.

### 2.5 Calculation of ACR

Several methods exist to calculate and infer ACR. A common method is to use real-time CO_2_ mixing ratios (or another tracer gas) and monitor decay rates.^65,66^ The work presented here used assumptions about the natural generation of CO_2_ by home occupants (adjusted for time spent in the main living area assessed through available data on room occupancy statistics^67,68^), the room volume, and the difference between internal and external CO_2_ mixing ratios. Within the wider scope of the INGENIOUS project, real-time CO_2_ mixing ratios were measured by low-cost sensors and used to calculate ACR, and these results will form the basis of a future paper.

Here, ACR was inferred according to an adapted method identified in Warburton *et al.* (2023),^14^ which itself used methods described by Batterman (2017),^69^ shown in eqn (1):1
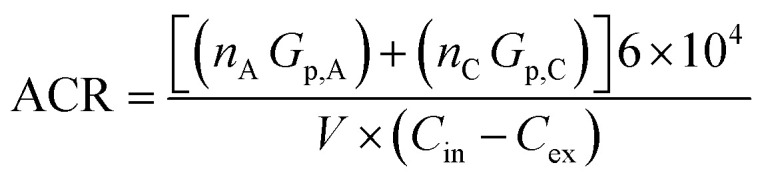
where ACR is the inferred air change rate (h^−1^), *n*_A_ and *n*_C_ is the number of adult and child occupants respectively, *G*_p,A_ and *G*_p,C_ are the natural generation rate of CO_2_ (L min^−1^) for adults and children respectively, *V* is the volume of the room the sample was taken in (m^3^), and *C*_in_ and *C*_ex_ are respectively the internal and external CO_2_ mixing ratios (ppm). The total room volume was calculated from measured room lengths, widths and heights. Volume was then reduced by 7% to account for volume occupied by internal furnishings, as in Manuja (2019),^70^ transforming the *V* term to be representative of available diluent volume. Here, *C*_ex_ was assumed to be 450 ppm. *G*_p,A_ and *G*_p,C_ used in this study were 0.312 L min^−1^ for an adult and 0.174 L min^−1^ for a child.^71^ Calculation by this method assumes the occupants were all present over the entire sampling period, which is unrealistic for up to 72 hours time-integrated sampling. Therefore, using available data on typical times spent in main living areas in the home, which was inclusive of time spent in other rooms in the home and working/school patterns, occupancy was adjusted to account for time not spent in the main living area.^67,68^ Clearly, assumptions have had to be made about external CO_2_ mixing ratios, occupancy patterns and CO_2_ generation rates in this approach, but the assumptions have been applied consistently across the data set based on knowledge of the number of occupants in each of the sampled houses.

### 2.6 VOC metrics and manipulation

For VOC analysis, a total of *n* = 124 samples were used. For each sample a metric of total VOC (“TVOC”) concentration was defined as the sum of all quantified VOC concentrations for each sample. This would often be a subset of the total number of VOCs quantified in this study, owing to variation in sample composition. Therefore, TVOC presented here is an operational air quality metric specific to this study and analytical method.

#### 2.6.1 Modified *Z*-score calculation

Modified *Z*-scores were calculated for VOCs for the analysis contained within Section 3.1.2. A ‘regular’ *Z*-score measures a value's deviation from the mean of the group it belongs to. In this study, it reflects how a VOC concentration deviates from the mean of all measured values for that VOC. A modified *Z*-score is similar but relies on the group median instead of the mean, making it less susceptible to skewing by outliers.

Indoor VOC sources such as paints and other decorating products can emit BTEX species (benzene, toluene, ethylbenzene, and xylene) and trimethylbenzenes (TMB) in intense but episodic bursts, especially immediately following the use of paints,^72^ potentially producing extreme outliers that distort data and statistical analyses. However, given the inherently variable nature of indoor air, a data treatment approach was needed that could accommodate natural variations while mitigating the impact of extreme values. Modified *Z*-scores were therefore calculated for indoor BTEX and TMB according to eqn (2):2
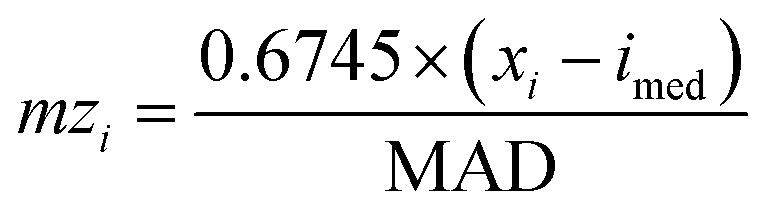
where *mz*_*i*_ is the modified *Z*-score, *x*_*i*_ is the value the modified *Z*-score is being calculated for (the VOC concentration for BTEX and TMB), *i*_med_ is the median of the group *i* to which *x*_*i*_ belongs (the median of all concentrations for the given BTEX or TMB VOC), and MAD is the median absolute deviation. Here, 
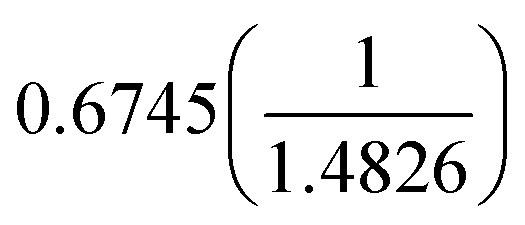
 acts to scale the modified *Z*-score to compare against ‘regular’ *Z*-scores. MAD itself is the median value of the absolute deviation of each data point in group *i* from the median of group *i*, and can be calculated according to eqn (3):

3 MAD = median(|*x*_*i*_ − *i*_med_|)

In this context, *mz*_*i*_ may be used analogously to a standard *Z*-score for evaluating deviations from the central tendency. However, because *mz*_*i*_ is based on group median and MAD, it is more resistant to the effects of outliers. Typically, if a value has an absolute *mz*_*i*_ greater than 3.5, it is said to be an outlier, and this threshold was used here.^73^

To address skewing by the largest outliers and reduce the number of false positives when identifying outliers through the *mz*_*i*_ calculation, the data were first logarithmically transformed. Outliers were then identified by calculating *mz*_*i*_ according to eqn (2), with datapoints grouped by VOC having an *mz*_*i*_ higher than 3.5 being filtered out. The typical lower threshold of −3.5 was not necessary in this case as the lowest modified *Z*-score for this analysis was −3.46. Finally, the data were transformed back to the original concentration values by applying an exponential to the filtered dataset.

#### 2.6.2 Time-averaged emission rate calculation

Time-averaged emission rates of VOCs from each sample were estimated by using measured indoor and outdoor concentrations, the internal room furnishing-adjusted room volume, and the inferred ACR for each sample. Time-averaged emission rates were calculated using a simple model adapted from Warburton *et al.* (2023),^14^ shown in eqn (4):4
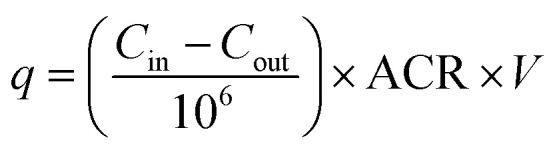
where *q* is the VOC time-averaged emission rate (g h^−1^), and *C*_in_ and *C*_out_ are the measured indoor and outdoor concentrations of the VOC respectively (μg m^−3^). In using eqn (4), it is assumed that the concentrations of the VOC is reflective of a steady-state value, and that VOC removal is a function solely of ventilation. The volume term (*V*) here reflected the 7% reduction of total room volume set out in Section 2.5. Eqn (4) does not give an instantaneous emission rate, rather a time-averaged emission rate of a VOC over the sampling period. While high VOC emission events will be captured by this methodology, they are smoothed given the low sampling flow rates. Sensitivity analysis for time-averaged emission rate calculations is given in supplementary method 1 (ESI†). The method for emission rate calculation here is mathematically equivalent to the methods of Sarwar *et al.* (2002).^74^

### 2.7 IRIS benchmark calculations

In this study, benzene exhibited a significantly lower median indoor concentration (0.70 μg m^−3^) compared to substances such as ethanol (median 320 μg m^−3^). Despite this, the health risks associated with chronic benzene overexposure are well-documented, with safe exposure thresholds set at notably low levels. Consequently, evaluating indoor concentrations against established exposure benchmarks offers additional insight into the potential long-term impacts of VOC exposure. Inhalation unit risks (IURs), defined as ‘the upper-bound excess lifetime cancer risk estimated to result from continuous exposure to an agent at a concentration of 1 μg m^−3^ in air for a lifetime’,^75^ for lifetime cancer risk (LCR) assessments and reference concentrations for hazard quotient (HQ) calculations were sourced from the IRIS database.^76^

Calculation of both LCR and HQ for homeowners follow methods established by both the United States Environmental Protection Agency (US EPA)^75,76^ and the Agency for Toxic Substances and Disease Registry (ATSDR),^77^ also using freely available data on UK working patterns obtained from the Office for National Statistics (ONS)^78^ to better inform assumptions made. These methods are given in the ESI as supplementary method 2 and 3† for LCR and HQ, respectively.

### 2.8 Data visualisation and statistical analysis

All data analysis and manipulation were conducted through *RStudio* software. The *tidyverse* package was used in all data processing. Data plotting used *ggplot2* for all figures. Boxplots show values in the order of (from bottom-to-top): lower outliers, 5th percentile, 25th percentile, median value, 75th percentile, 95th percentile, and upper outliers. Error bars on plots represent 95% confidence intervals, calculated using 1000 bootstrap resamples of the data.

Multiple statistical testing methods were employed in this study. Data normality was first assessed using QQ plots, which indicated that all VOCs followed non-normal distributions. To evaluate stochastic dominance within data subsets, Brunner–Munzel tests (also known as the “generalized Wilcoxon test”) were conducted, grouping samples based on a binary variable. The Brunner–Munzel test was chosen over the more commonly used Mann–Whitney *U* test (Wilcoxon rank-sum test) because it does not assume equal variances or a location shift between groups, unlike the Mann–Whitney *U* test. Given the variability of indoor air due to personal behaviours, these assumptions could not be reliably made, necessitating a more robust testing method.

For datasets with multiple levels within groupings (*e.g.*, seasons), an initial Kruskal–Wallis test was performed to assess overall differences across the primary binary grouping variable. *Post hoc* Dunn tests were then used to identify pairwise differences and determine specific levels exhibiting stochastic dominance.

All *p*-values were adjusted using Holm's correction for multiple comparisons. A significance level of *α* = 0.05 (95% confidence interval) was applied throughout the analysis, with *p*-values below this threshold indicating statistical significance.

## 3. Results and discussion

Raw VOC concentration and calculated time-averaged emission rate mean, quartile ranges and standard deviation is given in Tables S5 and S6 (ESI),† respectively.

### 3.1 Indoor VOC concentrations

#### 3.1.1 Indoor concentrations of select VOCs

**Fig. 1:**
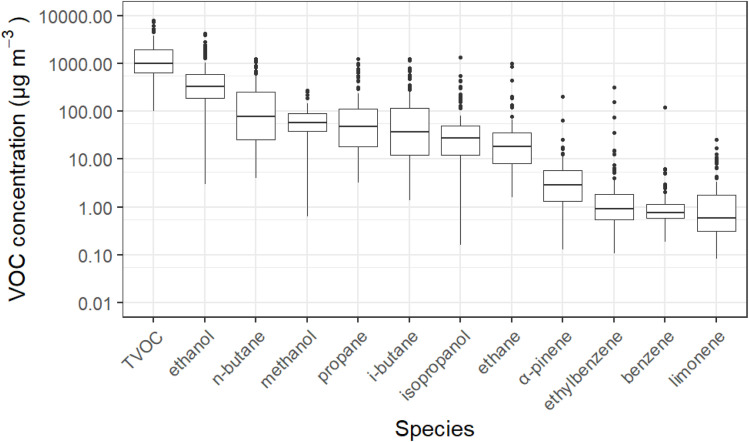
Measured indoor concentrations of a select group of VOCs measured in the INGENIOUS homes. Boxplots show values in the order of (from bottom-to-top): lower outliers, 5th percentile, 25th percentile, median value, 75th percentile, 95th percentile, and upper outliers. The *y*-axis has been logarithmically transformed to aid presentation.

Fig. 1 displays a logarithmic plot of TVOC and a selected group of VOCs found in indoor air samples in this study. The VOCs displayed in Fig. 1 were picked either due to their mass contribution to TVOC (methanol, ethanol, isopropanol, butane and propane), their inclusion as common ingredients in fragranced products (α-pinene and limonene), or being of note due to potential health concerns (benzene, ethylbenzene). On average across the 124 homes, ethanol, *n*-butane, methanol, propane and *i*-butane represented 11–93 wt% of the most abundant VOCs, with a median value of 68 wt%.

Indoor sources of butane and propane are dominated by emissions from compressed aerosol products such as deodorants, cleaning aerosols, hairsprays and other aerosolized personal care products (PCPs), where they act as propellants.^5^ Methanol is a commonly found emission from cooking, but also originates as an endogenous human breath emission, as well as in small amounts in some household products and, more rarely, PCPs.^16,79,80^ Ethanol is commonly found indoors, arising from multiple sources such as household products and PCPs, alcohol consumption and cooking.^3,16,79^

#### 3.1.2 Effect of geography on indoor VOC concentrations

**Fig. 2:**
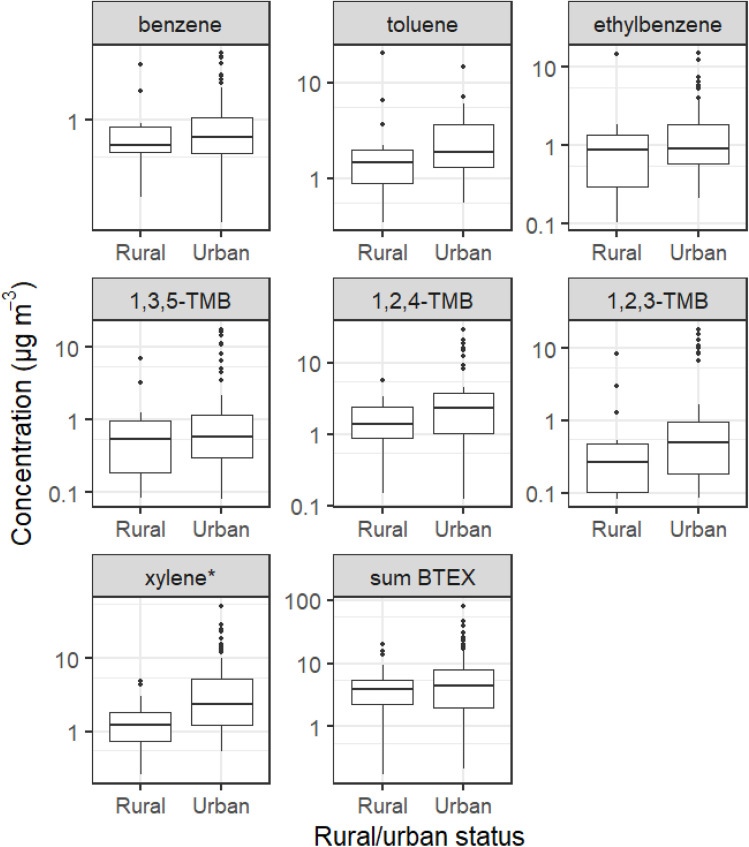
Indoor concentrations of aromatic VOCs, treated through modified *Z*-score analysis, and separated by rural or urban status. Boxplots show values in the order of (from bottom-to-top): lower outliers, 5th percentile, 25th percentile, median value, 75th percentile, 95th percentile, and upper outliers. The *y*-axis has been logarithmically transformed to aid presentation. TMB = trimethylbenzene, BTEX = benzene, toluene, ethylbenzene and xylene. * denotes a change of significance (*p*-value < 0.05).

Boxplots for indoor aromatic concentrations (once treated to account for extreme outliers) across rural and urban homes are shown in Fig. 2. The increase in indoor xylene concentrations between rural and urban homes was of significance (*p*-value = 0.02). There were modest but insignificant increases in median concentrations for 1,2,4- and 1,2,3-trimethylbenzene and toluene between rural and urban samples. There were minor increases in median benzene and ethylbenzene concentrations between rural and urban houses. Notably, urban homes exhibited a greater frequency of extreme concentration values compared to rural homes, further highlighting the trend that urban environments generally had higher concentrations of these species relative to rural settings. A similar analysis was applied to indoor/outdoor (I/O) ratios for these species (Fig. S2, ESI†), which were generally lower in urban compared to rural households, indicating that outdoor sources of these species were generally more prevalent in urban areas than in rural areas. An increase in indoor aromatic VOC concentrations in houses in urban areas, appeared to be driven by higher outdoor concentrations of aromatics.

#### 3.1.3 Lifetime cancer risk (LCR) estimates

**Fig. 3:**
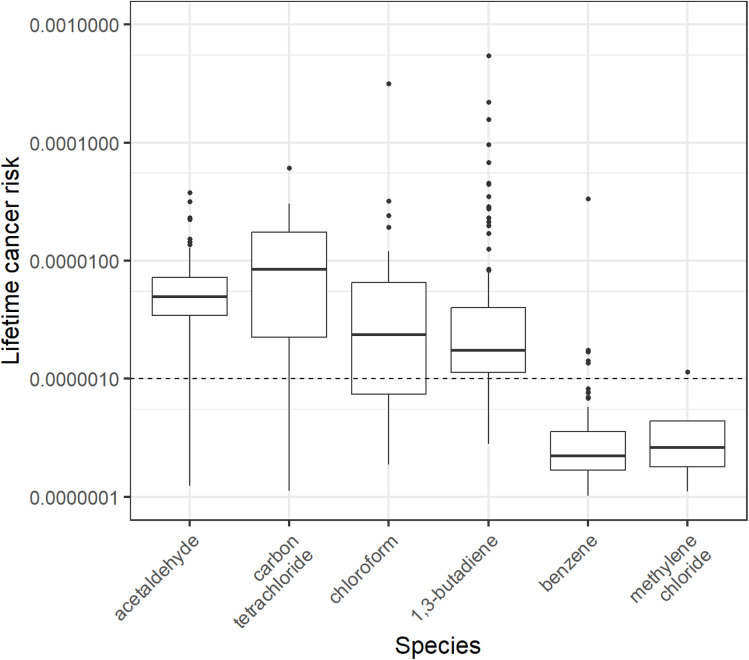
Calculated lifetime cancer risks (LCRs) for six VOCs. A horizontal dotted line is placed at the 0.000001 mark, representing the 1 in 1 000 000 chance threshold of developing cancer through a lifetime exposure to the exposure factor-adjusted concentration of the displayed VOC. Boxplots show values in the order of (from bottom-to-top): lower outliers, 5th percentile, 25th percentile, median value, 75th percentile, 95th percentile, and upper outliers. The *y*-axis has been logarithmically transformed to aid presentation.

Boxplots showing the spread of calculated LCRs for samples in this study are given in Fig. 3. A dashed horizontal line is shown at *y* = 1 × 10^−6^, which indicates a lifetime cancer risk of 1 in 1 000 000. Median values for acetaldehyde, carbon tetrachloride, chloroform and 1,3-butadiene were above the 1 in 1 000 000 threshold. Indeed, >60% of homes had concentrations of these four species above this threshold, with values sometimes exceeding 1 in 100 000, comparable to LCRs derived from chronic exposure to second- and third-hand smoke.^81,82^ However, LCRs in this study were highly variable and in general appeared to be somewhat lower than LCRs for chronic exposure to indoor airborne particulates and outdoor airborne nitrosamines.^82–85^ Carbon tetrachloride has been phased out of publicly available products since the Montreal Protocol came into effect, as well as being a potent hepatotoxic suspected human carcinogen. However given the variability in calculated LCRs and indoor concentrations, it is clear that there was at least one indoor source of emission of carbon tetrachloride in this study, likely arising as a secondary by-product of atmospheric reactions rather than as a primary emission from the product formulation itself. Carbon tetrachloride, as well as other halogenated hydrocarbons have been shown to be emitted from the use of chlorinated bleach indoors.^86,87^ The use of chlorinated products indoors may have been a potential source of indoor carbon tetrachloride secondary emissions here.

#### 3.1.4 Hazard quotients

**Fig. 4:**
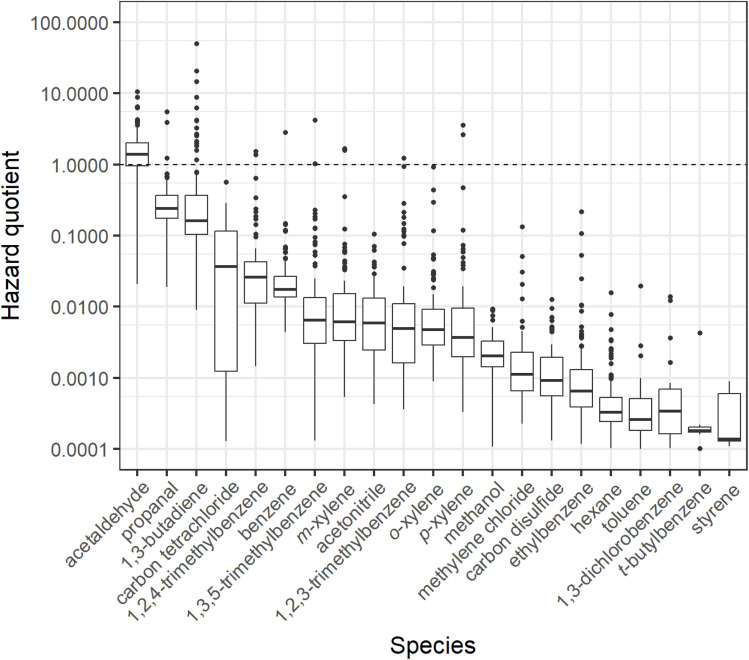
Calculated hazard quotients (HQs) for the VOCs measured in this study with available reference concentration data from the integrated risk information system (IRIS). A horizontal dotted line is placed at HQ = 1, above which a home occupant could develop symptoms based on a lifetime exposure to the exposure factor-adjusted concentration for the displayed VOC. Boxplots show values in the order of (from bottom-to-top): lower outliers, 5th percentile, 25th percentile, median value, 75th percentile, 95th percentile, and upper outliers. The *y*-axis has been logarithmically transformed to aid presentation.

Hazard quotients for all available VOCs are shown in Fig. 4. A dashed line was added at HQ = 1, as any value above this threshold is identified as exceeding the non-cancer health guidelines established by IRIS, defined as the threshold concentration above which a lifetime exposure risk not associated with cancer but other health outcomes is significantly possible.^76^Fig. 4 shows that concentrations for nine of the twenty-one analysed VOCs did not rise above the HQ = 1 threshold in any home. The highest number of total instances above LCR = 1 × 10^−6^ and HQ = 1 thresholds were for acetaldehyde and 1,3-butadiene. Most notable is the number of outlier observations in which 1,3-butadiene was markedly raised above LCR = 1 × 10^−6^ (*n* = 98 out of 124 total observations). Both acetaldehyde and 1,3-butadiene originate from, among other sources, wood products, combustion and use of cigarettes and e-cigarettes.^88–90^ Mean concentrations of acetaldehyde and 1,3-butadiene in this study were 22.2 μg m^−3^ and 3.6 μg m^−3^, respectively, placing mean indoor concentrations in Bradford homes for both species markedly higher than those found in literature.^3,14,88,91^

### 3.2 Air change rates

**Fig. 5:**
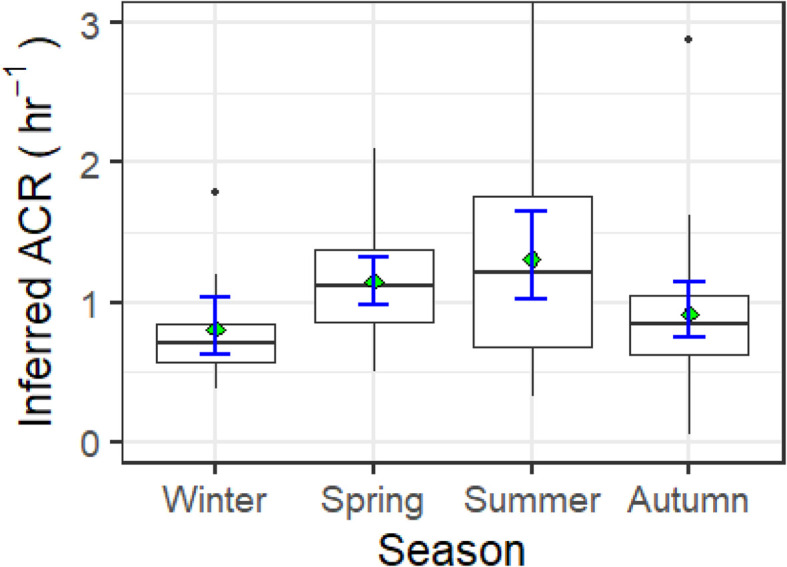
The inferred air change rates (h^−1^) by season. Boxplots show values in the order of (from bottom-to-top): lower outliers, 5th percentile, 25th percentile, median value, 75th percentile, 95th percentile, and upper outliers. The green diamonds indicate seasonal mean ACR. Error bars are calculated as 95% confidence intervals for the mean value using 1000 bootstrap resamples of the data.

Fig. 5 illustrates inferred ACR across the four seasons: winter (December, January, February), spring (March, April, May), summer (June, July, August), and autumn (September, October, November). The data reveal two distinct phases: an increase in ACR during spring and summer, followed by a decline in autumn and winter.

Meteorological data gathered from UK Met Office (Fig. S3(a) and (b), ESI†) shows that autumn had higher mean maximum (14.6 °C) and minimum (8.4 °C) temperatures in Bradford compared to spring (max mean 13.1 °C, min mean 5.2 °C). The gradual warming in spring, following the extended winter cold, likely heightened sensitivity to rising temperatures, prompting increased ventilation *via* open windows. Additionally, spring had more sunshine hours (mean 139.6 h per month) than autumn (mean 91.1 h per month), potentially leading to higher solar heating of buildings and warmer indoor temperatures, which may have contributed to the higher ACR in spring through windows and doors potentially being opened for longer.

Meteorological data for June 2023 indicated Bradford's highest mean temperature of the year (21.6 °C), while summer saw the most sunshine (mean 177 h per month). These meteorological patterns likely explain the elevated ACR observed in spring, which remained high throughout the summer as outdoor temperatures rose. The wider error bars for the mean summer ACR in Fig. 5 reflect greater variability in ACR during the summer months.

### 3.3 Seasonality in VOC emissions

Indoor VOC concentrations are primarily influenced by VOC emission rates, diluent room volume, and ACR. However, the observed seasonality in ACR suggested that raw indoor concentrations may not have fully captured the dynamics of indoor VOC exposure. Since VOC exposure is unique to the occupants of each sampled house, normalising concentrations by room volume and inferred ACR to calculate time-averaged VOC emission rates enables more robust comparisons across the cohort.

**Fig. 6:**
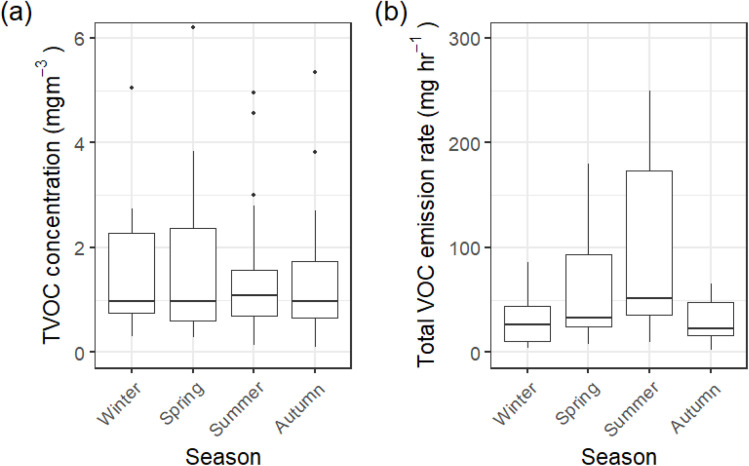
(a) Indoor TVOC concentrations by season, and (b) total indoor VOC emission rates by season. Boxplots show values in the order of (from bottom-to-top): lower outliers, 5th percentile, 25th percentile, median value, 75th percentile, 95th percentile, and upper outliers.

Fig. 6 shows a comparison between the seasonal TVOC concentrations in Fig. 6(a), with Fig. 6(b) showing seasonal total VOC time-averaged emission rates. There were no patterns in indoor TVOC concentration over the seasons (Fig. 6(a)), but clear seasonality in total indoor VOC emissions (Fig. 6(b)). Seasonality for individual VOC emissions is shown in Fig. S4, ESI.† In general, time-averaged emission rates were at a minimum in winter and a maximum in summer, as seen in total time-averaged emission rates in Fig. 6(b).

Total monoterpene time-averaged emission rates displayed the opposite seasonality however (Fig. S4, ESI†), with a low in summer and autumn months (median 492 μg h^−1^ and 380 μg h^−1^ respectively), and a high in winter and spring months (median 731 μg h^−1^ and 966 μg h^−1^ respectively). This was most likely driven by heightened emission rates of limonene in winter months, which was the single biggest contributor to the total monoterpene time-averaged emission rate metric. The reported elevated median use of fragrance products in winter months (Fig. S5, ESI†) is a likely source of elevated limonene emission rates in winter. A similar winter-high, summer-low pattern was seen in isopropanol, indicating the potential for common emission sources. However, emission sources are complex with variable dynamics, such as building materials which have variable rates of VOC off-gassing.^36,92^

As with indoor concentrations, indoor time-averaged emission rates were dominated by ethanol, butane and propane (Fig. S4, ESI†). However, the seasonality in time-averaged emission rates for these species does not match with the product use patterns (ESI Fig. S5†). It is noted that in a small subset of homes (*n* = 13), portable space heaters were used including those using bottled gas, which could be a non-typical source of indoor butane and propane. The use of bottled gas for cooking stoves is not common in the UK and was not found in this study. We further note that product use behaviours were consolidated in the questionnaires. For example, daily usage statistics of room fragrance products, such as air fresheners, electric diffusers and candles were grouped together. As a result, the grouping of product use behaviours provided a broader overview of behaviour, rather than allowing a highly specific breakdown of each product type.

Time-averaged emission rates of ethanol were markedly higher in summer months compared to others, with the summer median time-averaged emission rate (30 510 μg h^−1^) being more than three times the next highest median time-averaged emission rate in spring (9698 μg h^−1^). While inferred ACRs were higher in summer, there did not appear to be a significant seasonality in ethanol concentrations outdoors, and so higher outdoor air exchange in summer was unlikely to be the source of this trend.^93^

**Fig. 7:**
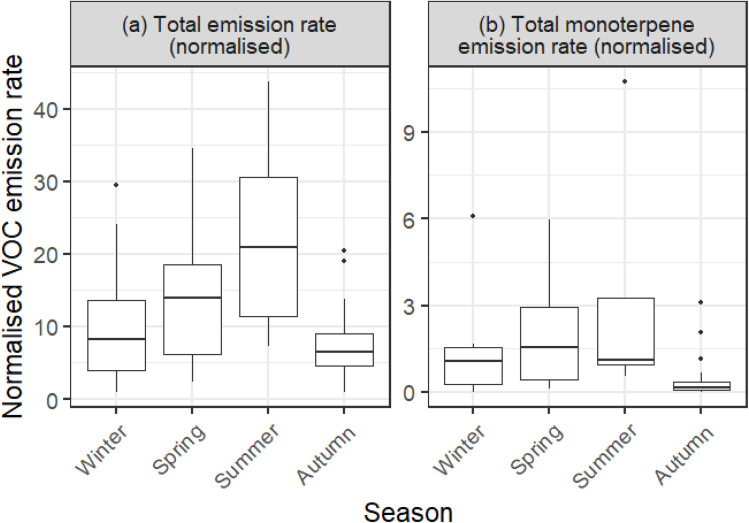
(a) Seasonality in the summed normalised total VOC emissions, and (b) the summed normalised monoterpene emissions. Boxplots show values in the order of (from bottom-to-top): lower outliers, 5th percentile, 25th percentile, median value, 75th percentile, 95th percentile, and upper outliers.

To account for single dominant time-averaged emission rates such as ethanol, propane and butane, VOC time-averaged emission rates were normalised on a scale of 0 to 1 using eqn (5), where 
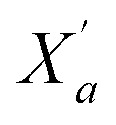
 is the normalised time-averaged emission rate value of VOC *a*, *X*_*a*_ is the original time-averaged emission rate of VOC *a* (g h^−1^), and *X*_*a*,max_ and *X*_*a*,min_ are the maximum and minimum time-averaged emission rates of VOC *a* (g h^−1^), respectively. Normalised values were then summed together, as shown in Fig. 7 along with summed normalised monoterpene time-averaged emission rates.5
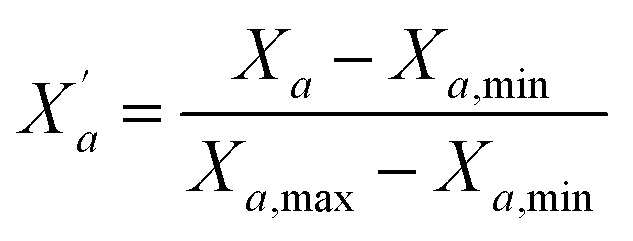


Total normalised VOC time-averaged emission rates were at their highest in summer and lowest in autumn. While Kruskal–Wallis testing on the non-normalised time-averaged emission rate data set indicated no significant change in total VOC time-averaged emission rates across the seasons, significance in total normalised VOC time-averaged emission rate and total normalised monoterpene time-averaged emission rate seasonality was observed. *Post hoc* Dunn tests revealed significant differences between autumn – summer and autumn – spring for both total normalised time-averaged emission rates and normalised monoterpene time-averaged emission rates. *p*-Values for the *post hoc* Dunn tests are displayed as a matrix in Fig. S6, ESI.† Normalised data analysis highlighted that dominance from specific VOC emissions could potentially skew results when comparing raw time-averaged emission rates. Among the possible sources of increased time-averaged emission rates in this study, two emerged as likely causes. Firstly, during the summer months, especially in late-July and August, children are at home more due to school holidays and as such, parents are likely to be at home more too. This could result in an increase in VOC emission rates. Secondly, it has been noted in other studies that emission rates of surface-partitioned VOCs increase with an increase in temperature.^17,94^ Over colder months, it has been suggested that there may be a cumulative increase in surface-bound VOC concentrations, which resulted in increased off-gassing as temperatures increase in warmer seasons.

## 4. Conclusions

The time-integrated concentrations of >120 VOCs were measured in the main living area of 124 homes in Bradford, UK. It was found, as in other studies,^3,14,95^ that indoor concentrations rarely showed any associations with factors such as product use. However, evaluation and estimation of personal VOC exposure using simple indoor concentrations against LCR benchmarks showed exceedances for all measured species against the US EPA 1 in 1 000 000 threshold, with exceedances in >75% of homes for acetaldehyde, carbon tetrachloride and 1,3-butadiene. HQ assessment showed an exceedance above an HQ of 1 in 75% of homes for acetaldehyde, and there were measured exceedances in HQ for propanal, 1,3-butadiene, trimethylbenzenes, benzene and xylenes. While not the aim of this paper to make direct claims regarding the health prospects of the participants of this study, there was a clear pattern of elevated concentrations of VOCs that have been shown to be harmful to health.

Of particular interest is carbon tetrachloride. As an ozone-depleting substance, as well as a hepatotoxic suspected human carcinogen, the inclusion of this VOC in any product has been phased out in many countries including the UK through the Montreal Protocol. However, the variation seen in this study (min 4.83 μg m^−3^, max 81 μg m^−3^, median 15.5 μg m^−3^) suggests that carbon tetrachloride has at least one secondary emission source indoors, likely as an atmospheric by-product of bleach or other chlorinated-product use. Previous studies have shown that carbon tetrachloride is a measurable emission from the use of bleach or other chlorinated-product use indoors.^86,87^ Further investigation into indoor production of carbon tetrachloride, other halogenated hydrocarbons and other VOCs from the use of household products would therefore be warranted.^44^

Once the effects of short-term emissions of BTEX species from painting and decorating had been accounted for, indoor BTEX species had higher median concentrations in urban homes than in rural homes, with xylene concentrations being significantly higher in urban homes (rural median concentration 1.22 μg m^−3^, urban median concentration 2.35 μg m^−3^). Additionally, there was a generally lower I/O ratio in urban houses for BTEX species compared with rural houses, indicating generally higher concentrations of BTEX species in urban outdoor areas. Indoor concentrations appeared to be impacted by outdoor concentrations with higher indoor concentrations for BTEX seen in urban homes compared to rural.

ACR was inferred using an adjusted method from Warburton *et al.* (2023)^14^ through CO_2_ exchange and room diluent volume. ACR was at a high in summer with a median ACR of 1.2 h^−1^ and at a low in winter with a median ACR of 0.7 h^−1^. ACR itself was most variable in summer, and presented a study-wide range of between 0.41 h^−1^ and 3.05 h^−1^.

Once seasonal changes in inferred ACR and individual room sizes were accounted for through the calculation of time-averaged emission rates, then highest VOC time-averaged emission rates were found for the summer months. Summer had the highest median total VOC time-averaged emission rate (51 950 μg h^−1^), while autumn had the lowest median total VOC time-averaged emission rate (22 760 μg h^−1^), closely followed by winter (26 161 μg h^−1^). Additionally, the variability in total VOC time-averaged emission rates rose to a maximum in summer (min 9472 μg h^−1^, max 249 300 μg h^−1^). This trend could not be attributed solely to any seasonality in product use in this study. This was likely a product of increased surface-adsorbed VOCs off-gassing as ambient temperature increased, as well as increased occupancy times over the summer months with parents and children spending more time in the homes.

### 4.1 Limitations and strengths

#### 4.1.1 Limitations

In this study, CO_2_ mixing ratios were used to infer an ACR. This clearly can have limitations since there may be unaccounted for sources of CO_2_ within indoor environments. While the samples from this study were time integrated thus smoothing short-term effects, this may have impacted the calculated ACR. In calculating time-averaged emission rates, only indoor and outdoor VOC concentrations, ACR and total room volume was considered. VOC loss through oxidation or surface losses may also have occurred, however accounting for these factors is not possible in a model given the state of knowledge of these processes at this time, and difficulties in estimating individual surface area-to-volume ratios and indoor oxidant concentrations for the large number of houses sampled here. Additionally, there remains little information available on surface deposition rates for many of the VOCs assessed here. Sensitivity analysis of the implications of assumptions based on ACR and room volume were completed and are included within the ESI.†

In calculating LCRs and HQs for indoor VOCs, assumptions must be made regarding expected lifetimes, residential times as well as a greater assumption that the sampled concentration of VOC is indicative of the concentration an occupant will always be exposed to. By nature, these calculations must make these assumptions, and the resulting values are only meant to be regarded as indicative values and not absolute.

Within the scope of the larger INGENIOUS study, participants answered several large questionnaires gathering information on many aspects of the occupants and their home. As such, aspects of the questionnaires had to be consolidated, such as product use. The resulting groupings were therefore relatively coarse.

#### 4.1.2 Strengths

A long-term analysis of indoor air quality has provided new insights into the multiple factors influencing indoor VOC exposure. By examining compounding seasonal effects and time-averaged emission rates, this study identifies key patterns in VOC variability. Previous research has shown that raw time-integrated VOC concentration data often fail to reveal meaningful relationships. However, through the application of transformative analytical methods, this study uncovers structured patterns in indoor VOC exposure. To our knowledge, this is one of the longest indoor VOC datasets collected to date.

This study offers a comprehensive dataset of indoor VOC concentrations and time-averaged emission rates across a large sample of homes (*n* = 124), serving as a valuable resource for the air pollution research community. The wide range of VOC species measured provides a detailed understanding of the factors shaping indoor air quality. By incorporating seasonal and spatial analyses, this work identifies key drivers of indoor VOC exposure, such as outdoor air ingress and emission variability throughout the year.

A key strength of this study is its contribution to understanding indoor VOC dynamics. For instance, the observed increase in ACR during warmer months suggests greater ventilation-driven transport of VOCs from indoors to the immediate outdoor environment. When scaled across millions of homes, this process may represent a significant and largely unaccounted-for source of outdoor VOC pollution. Currently, most outdoor air pollution models do not adequately consider indoor VOC emissions, yet this study demonstrates that indoor spaces could play a major role in shaping outdoor VOC concentrations.

### 4.2 Future work

Further in-home studies should attempt to specify product use as much as possible. The realities of widely variable product formulation and composition will naturally result in assumptions having to be made when considering source apportionment of VOCs to specific groups of products, however. Future indoor air analyses should consider the effects of ACR and diluent room volume on indoor VOC concentrations. While indoor concentrations can be used to compare against benchmarks, the effects of intra-study ACR and volume variability on measured concentrations may result in difficulty in drawing conclusions from concentration data across and within studies. Compounding this with sampling and analytical differences between studies, transformation of indoor concentrations into emission rates may allow for better VOC exposure comparison between studies. Additional consideration of the potential for increased VOC-surface partitioning in colder months and increased off-gassing of surface-bound VOCs in warmer months would provide additional insight into population exposure to VOCs.

Future studies should also consider the impact indoor air pollution may have on outdoor air pollution. As is evident from this study, indoor VOC concentrations can be orders of magnitude higher than outdoor concentrations, and activities such as cooking and cleaning are known to give rise to substantial VOC emissions. Indoor reactions appear capable of producing halocarbons of significance to stratospheric ozone depletion such as carbon tetrachloride, species that are not used as raw ingredients in product formulation, but may still be emitted due to unaccounted for indoor chemistry. Mitigating indoor VOC exposure by increased ventilation will directly lead to elevated outdoor VOC concentrations in the immediate surroundings of the indoor area, and the magnitude of this effect should be further studied and quantified.

## Data availability

All sampled data from this study is freely available from https://doi.org/10.15124/24fd1762-0e98-4773-a74c-7dd87ef59aa8.

## Authors contribution

TW – methodology, investigation, visualisation, data curation, formal analysis, validation, writing-original draft, writing-review & editing. ACL – conceptualisation, methodology, writing-review & editing, funding acquisition. JFH – conceptualisation, methodology, writing-review & editing, funding acquisition. RMCM – conceptualisation, methodology, investigation, writing-review & editing, funding acquisition. TCY – methodology, investigation, writing-review & editing. JRH – methodology, writing-review & editing. SJA – methodology, writing-review & editing. NC – conceptualisation, methodology, writing-review & editing, funding acquisition.

## Conflicts of interest

There are no conflicts of interest to declare.

## Supplementary Material

EM-027-D4EM00756E-s001
