# Supplementary material for: Yearlong study of indoor VOC variability: insights into spatial, temporal, and contextual dynamics of indoor VOC exposure
Source: Environ Sci Process Impacts. 2025 Mar 12;27(4):1025–40. doi: 10.1039/d4em00756e (PMC11921335; doi:10.1039/d4em00756e)
Supplement: EM-027-D4EM00756E-s001 [file EM-027-D4EM00756E-s001.pdf]

This journal is © The Royal Society of Chemistry 2025

Supplementary information (SI)

Fig. S1

The extent of the clean air zone (CAZ), as well as a topographical presentation of the elevations surrounding Bradford. Green represents low heights above sea level, increasing up to red. The CAZ boundary (shown as a red outline) is flanked by areas of higher ground, increasing up to the Pennine hills in West Bradford.

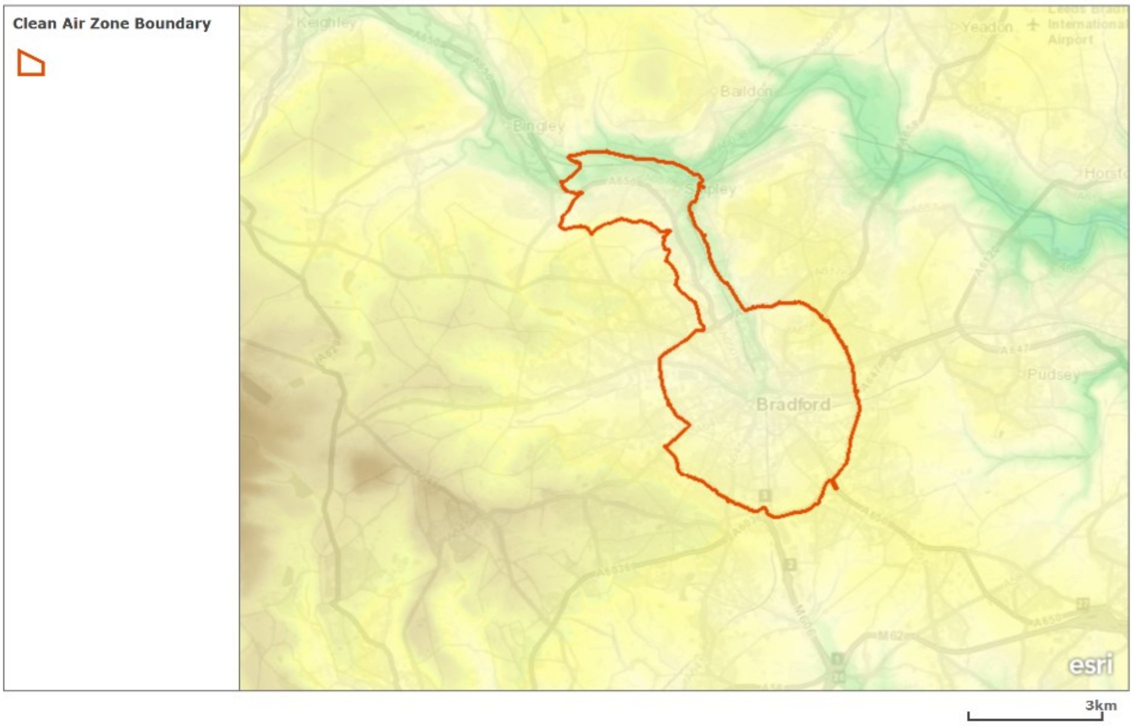

Contains OS data © Crown Copyright and database right 2016 | Contains OS data © Crown Copyright and database right 2020

Table S1

A table of the 30 VOCs contained within the 30-component NPL30 calibration standard used in this study.

1,2,3-trimethylbenzene	ethane	n-heptane
1,2,4-trimethylbenzene	ethene	n-octane
1,3,5-trimethylbenzene	ethylbenzene	n-pentane
1,3-butadiene	hexane	o-xylene
1-butene	isobutane	propane
2-methylpentane	isooctane	propene
acetylene	iso-pentane	p-xylene
benzene	isoprene	toluene
cis-2-butene	m-xylene	trans-2-butene
cis-2-pentene	n-butane	trans-2-pentene

Table S2

Limits of detection (LOQ) and quantification (LOQ) for VOCs resolved by the GC analysis. LODs were calculated by a signal-to-noise (SNR) ratio of 3:1, and LOQ using an SNR of 10:1.

voc_name	LOD_µgm3	LOQ_µgm3	voc_name	LOD_µgm3	LOQ_µgm3
123-TMB	0.0217	0.0724	gamma-terpinene	0.0227	0.0755
124-TMB	0.0222	0.0741	hexane	0.0239	0.0796
135-TMB	0.0222	0.0741	isobutane	0.0242	0.0806
2-carene	0.0227	0.0755	isoprene	0.0227	0.0755
2-chloropropane	0.0436	0.145	isopropanol	0.0444	0.148
3-carene	0.0227	0.0755	levomenthol	0.0281	0.0937
4-carene	0.0227	0.0755	limonene	0.0227	0.0755
acetaldehyde	0.0733	0.244	methanol	0.0711	0.237
acetone	0.0483	0.161	methylenechloride	0.141	0.471
acetonitrile	0.0455	0.152	n-butane	0.0242	0.0806
alpha-phellandrene	0.0227	0.0755	n-pentane	0.024	0.08
alpha-pinene	0.0227	0.0755	o-cymene	0.0223	0.0744
alpha-terpinene	0.0227	0.0755	p-cymene	0.0223	0.0744
beta-myrcene	0.0227	0.0755	propan-1-ol	0.04	0.133
beta-phellandrene	0.0227	0.0755	propanal	0.0483	0.161
beta-terpinene	0.0227	0.0755	propane	0.0245	0.0815
beta-thujene	0.0227	0.0755	styrene	0.0217	0.0722
camphene	0.0227	0.0755	tert-butyl-benzene	0.0223	0.0744
camphor	0.0281	0.0938	11-dichloroethene	0.101	0.336
carbondisulfide	0.127	0.422	cyanogenchloride	0.128	0.426
delta-terpinene	0.0227	0.0755	carbontetrachloride	1.02	3.41
ethanol	0.0511	0.17	chloroform	0.496	1.65
ethylbenzene	0.0221	0.0736	benzene	0.0217	0.0722
toluene	0.0219	0.073	xylene	0.0221	0.0736

Supplementary method 1 – emission rate sensitivity analysis

Emission rate in this paper was calculated as in eqn SE1:

$$q = (C_{in} - C_{out}) \times V \times ACR$$

SE1

Where q is the calculated emission rate (g hr^{-1}), C_{in} and C_{out} are the indoor and outdoor concentration of a VOC (µg m^{-3}), V is the available diluent volume (m^3) and ACR is the air change rate (hr^{-1}). To assess the impact of compounding errors on the emission rate, the propagation of errors formula can be used:

$$\sigma_q^2 = \left(\frac{\partial q}{\partial C_{in}}\sigma_b\right)^2 + \left(\frac{\partial q}{\partial C_{out}}\sigma_c\right)^2 + \left(\frac{\partial q}{\partial V}\sigma_d\right)^2 + \left(\frac{\partial q}{\partial ACR}\sigma_e\right)^2$$

SE2

Where $\frac{\partial q}{\partial x}$ is the partial derivative of the emission rate q with respect to variable x, and σ_x is the absolute error of variable x. The partial derivatives can be calculated using the product rule as such:

$$\frac{\partial q}{\partial C_{in}} = V \times ACR$$

$$\frac{\partial q}{\partial C_{out}} = - V \times ACR$$

$$\frac{\partial q}{\partial V} = (C_{in} - C_{out}) \times ACR$$

$$\frac{\partial q}{\partial ACR} = (C_{in} - C_{out}) \times V$$

This then gives the final equation in SE3:

$$\sigma_q = \sqrt{\left(V \times ACR \times \sigma_{C_{in}}\right)^2 + \left(- V \times ACR \times \sigma_{C_{out}}\right)^2 + \left((C_{in} - C_{out}) \times ACR \times \sigma_V\right)^2 + \left((C_{in} - C_{out}) \times V \times \sigma_{ACR}\right)^2}$$

SE3

The errors of C_{in} and C_{out} are 0.1%, which itself was determined through constructing calibration curves of a target gas of increasing concentration. As conservative estimates, errors in V and ACR were assigned as 10% and 20%, respectively. This resulted in an average 22% uncertainty in the calculated emission rate. However, this likely represents a 'worst case' scenario as the volume and air change rate uncertainties are likely upper-bound estimates of the actual uncertainties. Assigning errors in V and ACR of 10% for both gives as average 14% uncertainty in the calculated emission rate.

Supplementary method 2 – Lifetime cancer risk calculation

Lifetime cancer risk (LCR) can be calculated according to eqn SE4:

$$LCR = C_{A,C} \times IUR$$

SE4

Where $C_{A,C}$ is the exposure factor-adjusted indoor concentration (μg m^{-3}), T_Y is the residential time per lifetime (years), and T_L is the life expectancy (years), and IUR is the

inhalation unit risk [(μg m^{-3})$^{-1}$]. Cancer IURs were identified for 6 VOCs in this study, and were obtained from the Integrated Risk Information System (IRIS) database, shown in Table S4, hosted by the United States Environmental Protection Agency (US EPA).[1] The EPA defines the IUR as 'the upper-bound excess lifetime cancer risk estimated to result from continuous exposure to an agent at a concentration of 1 μg m^{-3} in air for a lifetime'. [2]

The exposure factor-adjusted indoor concentration was calculated using eqn SE5:

$$C_{A,C} = C_I \times E_{f,C}$$

SE5

Where C_I is the measured indoor concentration obtained from whole air samples (μg m^{-3}), and $E_{f,C}$ is the atmospheric exposure factor for the LCR calculation (dimensionless). The atmospheric exposure factor serves to adjust the measured indoor concentrations into an 'effective' concentration to account for time not spent within the setting C_I was measured in.

$E_{f,C}$ was calculated according to eqn SE6:

$$E_{f,C} = \frac{T_R \times 7\frac{days}{week} \times 52.14\frac{weeks}{year} \times T_Y}{24\frac{hours}{day} \times 7\frac{days}{week} \times 52.14\frac{weeks}{year} \times T_L}$$

SE6

Where T_R is the residential time per day (hours), T_Y is the residential time per lifetime (years), and T_L is the life expectancy (years). Average UK work hours (obtained from the Office for National Statistics [3]) were subtracted from 24 to give $T_R = 16.72.$ Values of

$T_Y = 33$ and $T_L = 78$ were obtained from available guidance provided by the Agency for Toxic Substances and Disease Registry (ATSDR). [4] This gave a calculated $E_{f,C}$ of 0.30, reflecting that for this calculation it is assumed that 30% of an individual's lifetime is spent in the residential environment studied here,

Supplementary method 3 - Hazard quotient calculation

The calculation of a hazard quotient (HQ) for a VOC concentration starts similarly to the estimation of LCR in the calculation of an exposure factor, shown in eqn SE7:

$$E_{f,NC} = \frac{T_R \times 7\frac{days}{week} \times 52.14\frac{weeks}{year}}{24\frac{hours}{day} \times 7\frac{days}{week} \times 52.14\frac{weeks}{year}}$$

SE7

Where $E_{f,NC}$ is the atmospheric exposure factor for non-cancer hazard quotient calculation (dimensionless). $E_{f,NC}$ in this study was calculated to be 0.70. The exposure factor-adjusted concentration of VOCs for non-cancer hazard quotient calculation, $C_{A,NC}$ was calculated as in eqn SE5. Finally, hazard quotients were calculated as in eqn SE8:

$$HQ = \frac{C_{A,NC}}{R_f C}$$

SE8

Where HQ is the hazard quotient for non-cancer related risk (dimensionless), and $R_f C$ is the reference concentration for each VOC (μg m^{-3}). $R_f C$ values were gathered from the IRIS database, and are shown in Table S5. [1] The preceding equations follow guidance from the EPA and the ATSDR. Cancer IURs were calculated using specific exposure

pathways, as inhalation risks increase through defined routes. In contrast, R_fC values consider total indoor time, reflecting broader exposure patterns in the calculation of exposure factors.

Table S3

Measured VOCs used for LCR analysis using inhalation unit risk data available from IRIS.

**Species name**	**Inhalation unit risk (per μg m^{-3})**
1,3-butadiene	0.00003
Chloroform	0.000023
Carbon tetrachloride	0.000006
Acetaldehyde	0.0000022
Benzene	0.0000022
Methylene chloride	0.00000008

Table S4

Measured VOCs used for HQ analysis using reference concentration data available from IRIS.

**Species name**	**Reference concentration (μg m^{-3})**
1,3-butadiene	2
Propanal	8
Acetaldehyde	9
Benzene	30
1,2,3-trimethylbenzene	60
1,2,4-trimethylbenzene	60
1,3,5-trimethylbenzene	60
Acetonitrile	60
m-xylene	100
o-xylene	100
p-xylene	100
Methylenechloride	600
Carbondisulfide	700
Hexane	700
1,3-dichlorobenzene	800
Ethylbenzene	1000

Styrene	1000
t-butyl-benzene	5000
Toluene	5000
Methanol	20000
Carbon tetrachloride	100
Cyclohexane	6000
Naphthalene	3
t-butyl-alcohol	5000

Table S5

Raw sampled VOC concentration data. All values are given in units of µg m^{-3}.

Species	Mean	5th percentile	25th percentile	Median	75th percentile	95th percentile	Standard deviation
propane	131.4718	6.42451	18.22264	49.01542	113.2612	620.9195	216.4602
isobutane	128.7174	2.869592	12.18533	37.30631	117.3661	678.1206	236.4613
n-butane	219.6981	6.66134	25.96672	76.44349	250.0136	1101.071	317.5535
n-pentane	4.286987	0.487911	1.189555	1.932624	3.791783	11.53045	10.04894
hexane	0.770075	0.117996	0.230816	0.322301	0.52026	2.557107	1.70827
isoprene	2.113299	0.3898	0.769828	1.541363	2.807606	5.192791	2.090033
carbondisulfide	1.862036	0.451615	0.637903	1.072044	1.99231	6.308651	2.15951
acetaldehyde	22.41466	1.549082	12.3473	18.10759	26.00685	49.93836	19.36909
propanal	4.247747	0.466236	1.99725	2.795617	4.234733	8.406247	7.263306
acetone	53.50257	10.58774	25.38392	32.89584	54.54952	200.9483	70.68356
methanol	69.86539	3.161335	38.55206	57.75694	89.92292	144.7383	50.60594
methylenechloride	5.384164	0.507537	0.784996	1.272674	2.361142	21.32572	16.8556
propan-1-ol	0.554775	0.1396	0.189485	0.257577	0.493002	1.134415	0.993939
isopropanol	72.65894	1.53941	12.4102	27.6249	49.88171	316.2054	156.09
ethanol	558.9503	68.56741	191.8341	332.4858	591.9274	1942.875	708.2073
4-carene	2.879976	0.146486	0.804301	2.224512	4.935448	6.773487	2.521787
acetonitrile	1.14215	0.180395	0.319073	0.675818	1.294433	3.441378	1.417328
alpha-pinene	6.75427	0.33136	1.350052	2.923961	5.795429	13.24767	20.76261
beta-myrcene	1.246831	0.085531	0.172666	0.450656	1.173801	4.84582	2.571431
alpha-terpinene	0.858637	0.08269	0.145405	0.227314	0.420593	1.789995	3.413624
tert-butyl-benzene	1.773789	0.095468	0.136105	0.448511	1.15561	1.538526	6.106573
135-TMB	6.874903	0.116183	0.294255	0.578319	1.237453	15.39759	38.66844
124-TMB	7.385853	0.152064	0.960881	2.278751	3.712229	22.76234	21.02322

123-TMB	3.489957	0.093109	0.172943	0.463392	0.980196	12.66378	13.44574
styrene	0.342512	0.076227	0.115811	0.158484	0.198685	1.114931	0.429401
camphene	0.183171	0.079062	0.09358	0.136743	0.203193	0.509951	0.138601
beta-thujene	1.021399	0.105891	0.178882	0.379276	1.188809	3.149632	1.615707
beta-terpinene	1.383296	0.137074	0.635795	1.360244	2.132334	2.662272	0.989501
3-carene	0.874588	0.088192	0.177476	0.410748	0.861806	2.541324	1.657697
alpha-phellandrene	0.144441	0.099342	0.106019	0.140034	0.178455	0.195707	0.049214
limonene	2.050206	0.113666	0.314121	0.58791	1.774617	9.888409	3.874785
beta-phellandrene	1.228936	0.08996	0.120912	0.193715	1.130563	3.222927	2.483957
gamma-terpinene	0.31083	0.085268	0.126395	0.183131	0.398071	0.871774	0.296988
delta-terpinene	0.612528	0.078937	0.109944	0.119627	0.280612	2.176802	1.671658
2-carene	2.989718	0.22764	0.577392	1.18232	1.400473	11.44858	5.610187
levomenthol	0.549449	0.111394	0.175855	0.337634	0.41317	1.927401	0.699965
camphor	0.558731	0.200694	0.21291	0.437213	0.666318	1.311859	0.480022
o-cymene	0.608298	0.085367	0.092742	0.11699	0.185033	1.835551	1.953696
p-cymene	0.704738	0.103167	0.200084	0.408544	0.893663	2.162917	0.723335
ethylbenzene	6.679928	0.25514	0.546938	0.923522	1.859595	12.98793	32.88894
carbontetrachloride	21.0364	5.168289	10.85679	15.46533	31.3838	39.76674	16.63856
chloroform	8.761406	1.731696	1.857239	2.456674	3.816694	16.05265	23.87512

Table S6

Calculated emission rate data in units of g hr^{-1}.

Species	Mean	5th percentile	25th percentile	Median	75th percentile	95th percentile	Standard deviation
propane	0.00525	6.93E-05	0.000408	0.00168	0.003809	0.023865	0.009542
isobutane	0.004476	2.85E-05	0.000299	0.000855	0.003463	0.021851	0.008269
n.butane	0.009971	9.20E-05	0.000792	0.001937	0.008838	0.043268	0.018425
n.pentane	9.47E-05	2.75E-06	1.43E-05	4.01E-05	9.02E-05	0.000432	0.000165
isoprene	8.12E-05	8.84E-06	2.56E-05	5.73E-05	9.40E-05	0.000249	8.97E-05
acetone	0.001725	0.000214	0.00055	0.000927	0.001479	0.005462	0.002365
methanol	0.002165	0.000285	0.000768	0.001486	0.002752	0.00541	0.00247
methylenechloride	8.94E-05	3.65E-06	1.13E-05	3.01E-05	5.71E-05	0.000437	0.000199
propan.1.ol	1.12E-05	9.17E-07	2.30E-06	5.70E-06	1.04E-05	4.01E-05	1.85E-05
isopropanol	0.00263	0.000245	0.000553	0.001158	0.002354	0.011096	0.004334
ethanol	0.014077	0.001371	0.004283	0.008894	0.018304	0.037413	0.015768
4.carene	7.73E-05	8.46E-09	8.86E-08	3.48E-05	0.000137	0.000216	0.000106
alpha.pinene	0.000166	7.90E-06	3.81E-05	9.30E-05	0.000232	0.0005	0.000182
beta.myrcene	1.57E-05	7.32E-07	2.26E-06	5.81E-06	1.03E-05	2.48E-05	5.08E-05
alpha.terpinene	1.41E-05	2.19E-06	3.98E-06	7.30E-06	1.27E-05	4.28E-05	2.22E-05
135.TMB	3.55E-05	2.50E-06	7.42E-06	1.82E-05	2.98E-05	7.95E-05	8.98E-05
124.TMB	0.000163	4.97E-06	2.37E-05	6.24E-05	0.0001	0.000817	0.000375
123.TMB	2.68E-05	1.95E-06	4.18E-06	8.51E-06	2.06E-05	6.00E-05	9.98E-05
camphene	3.19E-06	4.34E-09	2.49E-07	1.44E-06	3.31E-06	9.27E-06	6.19E-06
beta.thujene	3.72E-06	1.03E-08	2.27E-07	1.29E-06	5.23E-06	1.08E-05	6.27E-06
beta.terpinene	1.41E-06	1.99E-08	9.79E-08	2.38E-07	3.79E-07	5.37E-06	4.58E-06
3.carene	4.67E-05	4.47E-07	6.01E-06	1.74E-05	4.20E-05	0.000222	8.58E-05
alpha.phellandrene	1.23E-06	2.12E-08	1.52E-07	6.87E-07	1.50E-06	3.92E-06	1.86E-06
limonene	9.37E-05	9.45E-07	8.29E-06	2.70E-05	0.000114	0.000392	0.000147

beta.phellandrene	4.47E-06	7.34E-09	7.99E-08	1.29E-06	4.74E-06	2.38E-05	7.68E-06
alpha.terpinene	1.29E-06	9.83E-08	2.47E-07	3.65E-07	5.97E-07	3.27E-06	3.38E-06
gamma.terpinene	8.00E-06	1.11E-07	6.81E-07	3.57E-06	1.04E-05	2.10E-05	1.75E-05
delta.terpinene	1.27E-05	6.98E-09	1.94E-07	9.96E-07	2.54E-06	1.08E-05	7.83E-05
2.carene	1.36E-06	2.29E-08	1.18E-07	2.47E-07	5.95E-07	6.38E-06	3.35E-06
levomenthol	1.72E-06	1.42E-07	2.56E-07	4.11E-07	2.55E-06	4.97E-06	2.42E-06
o.cymene	2.66E-06	2.90E-07	5.44E-07	1.39E-06	4.66E-06	7.41E-06	2.70E-06
p.cymene	3.39E-05	8.73E-07	4.39E-06	1.87E-05	3.87E-05	0.000102	5.33E-05
2.chloropropane	4.40E-06	8.71E-08	1.63E-07	2.37E-07	4.33E-06	1.64E-05	7.96E-06
ethylbenzene	0.000124	3.23E-06	1.06E-05	2.73E-05	5.72E-05	0.000162	0.000625
ethylbenzene	0.00015	6.59E-06	8.56E-06	1.48E-05	2.61E-05	0.000161	0.000588
carbontetrachloride	1.52E-05	5.52E-07	1.28E-06	3.70E-06	1.44E-05	7.40E-05	2.65E-05
chloroform	2.22E-05	2.97E-06	4.79E-06	1.39E-05	3.44E-05	5.91E-05	1.99E-05

Fig. S2

Boxplots indoor/outdoor ratios of aromatic VOCs, separated by rural or urban status. Indoor/outdoor ratios were split into Boxplots show values in the order of (from bottom-to-top): lower outliers, 5th percentile, 25th percentile, median value, 75th percentile, 95th percentile, and upper outliers. TMB = trimethylbenzene. Here, individual xylene isomers have been grouped for consistency with the main text.

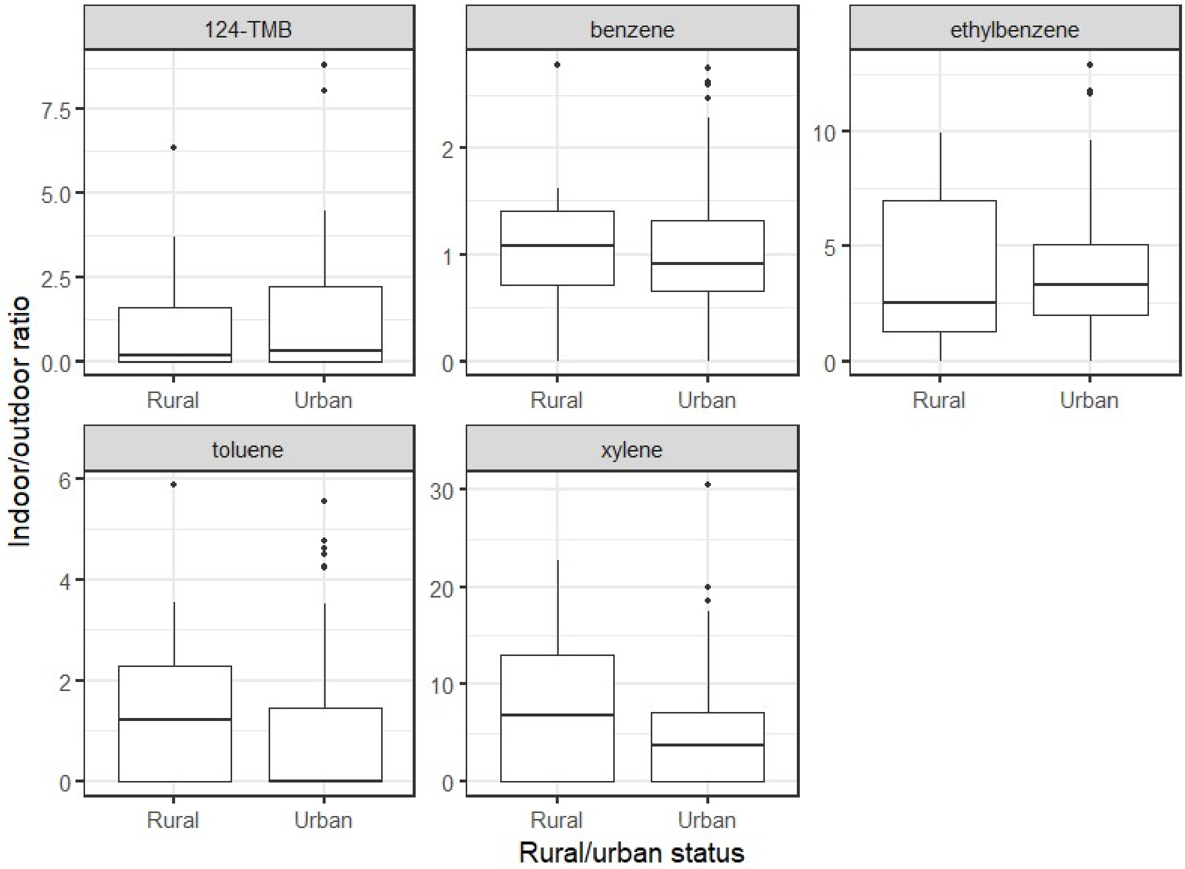

Fig. S3(a)

Meteorological data gathered from UK Met Office for Bradford over 2023 and 2024, taken from a weather station at 53°48'46.8"N 1°46'19.2"W. Values were taken for each month. Data was collected for maximum and minimum mean temperature (^{O}C), ground

frost days per month, total rainfall (mm) and total sunshine hours per month.

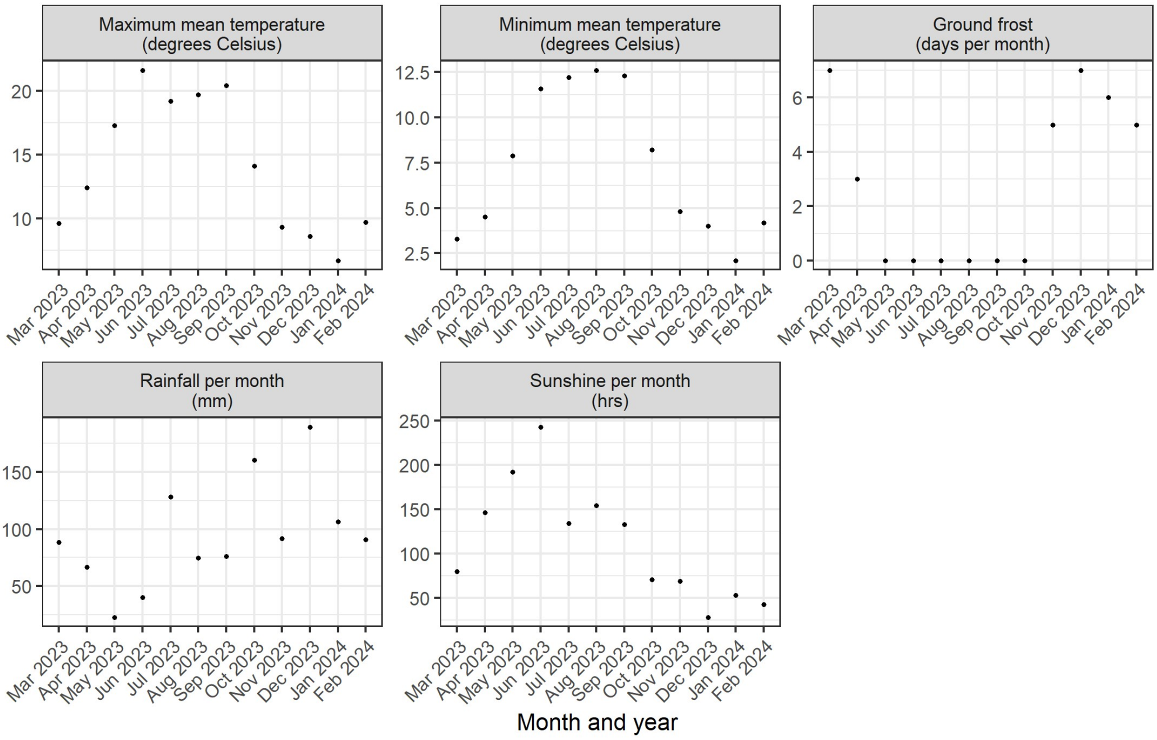

Fig. S3(b)

Meteorological data gathered from UK Met Office for Bradford over 2023 and 2024, taken from a weather station at 53°48'46.8"N 1°46'19.2"W. Data was collected for maximum and minimum mean temperature (^{O}C), ground frost days per month, total rainfall (mm) and total sunshine hours per month. Values were taken for each month, with each month then being grouped into four seasons: winter (December, January, February), spring (March, April, May), summer (June, July, August), and autumn (September, October, November), and mean values for each meteorological measurement taken for each season.

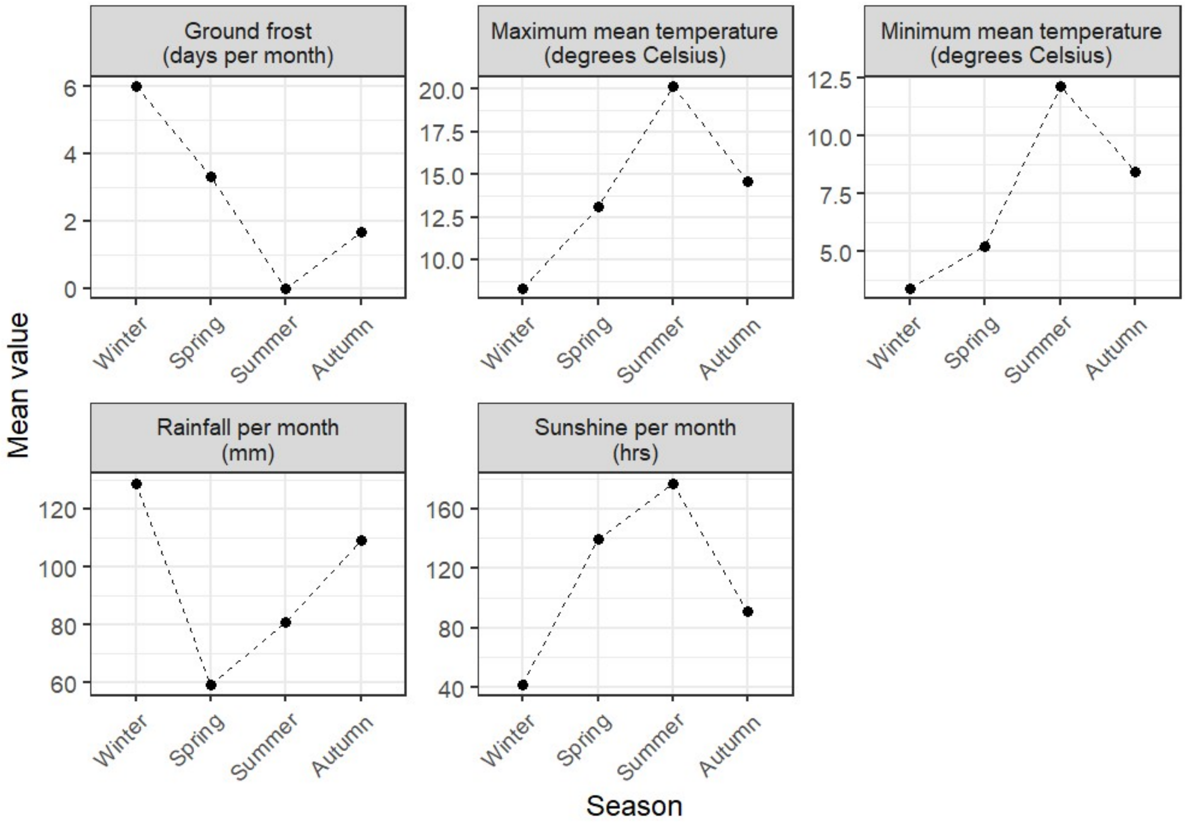

Ground frost
(days per month)
Maximum mean temperature
(degrees Celsius)
Minimum mean temperature
(degrees Celsius)
Rainfall per month
(mm)
Sunshine per month
(hrs)
Mean value
Season
Winter
Spring
Summer
Autumn

Fig. S4

Calculated individual VOC emission rates over the seasons. Outliers above 10 mg hr^{-1} were removed from ethane and sum BTEX plots, and outliers above 1 mg hr^{-1} were removed from 1,3-butadiene and sum TMB plots to aid presentation, but did not affect the calculation of quartiles for boxplots. Boxplots show values in the order of (from bottom-to-top): lower outliers, 5th percentile, 25th percentile, median value, 75th percentile, 95th percentile, and upper outliers. The y-axis has been logarithmically transformed to aid presentation. TMB = trimethylbenzene, BTEX = benzene, toluene, ethylbenzene and xylene.

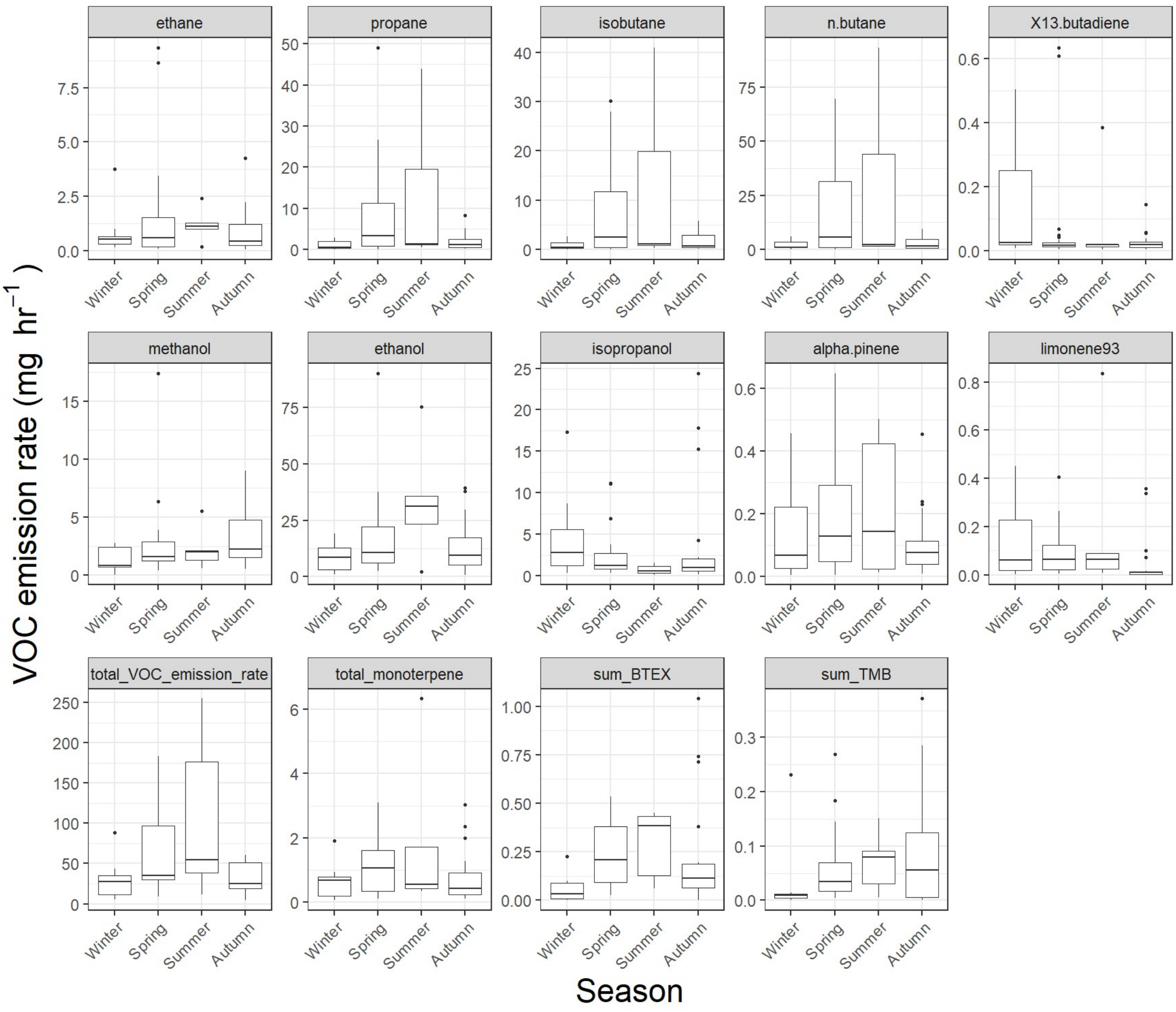

Fig. S5

(a) Total fragrance product use, (b) total aerosol product use and (c) the sum of fragrance and aerosol product use. Statistics were gathered daily and then added together over the 72-hour sampling period. Boxplots show values in the order of (from bottom-to-top): lower outliers, 5th percentile, 25th percentile, median value, 75th percentile, 95th percentile, and upper outliers.

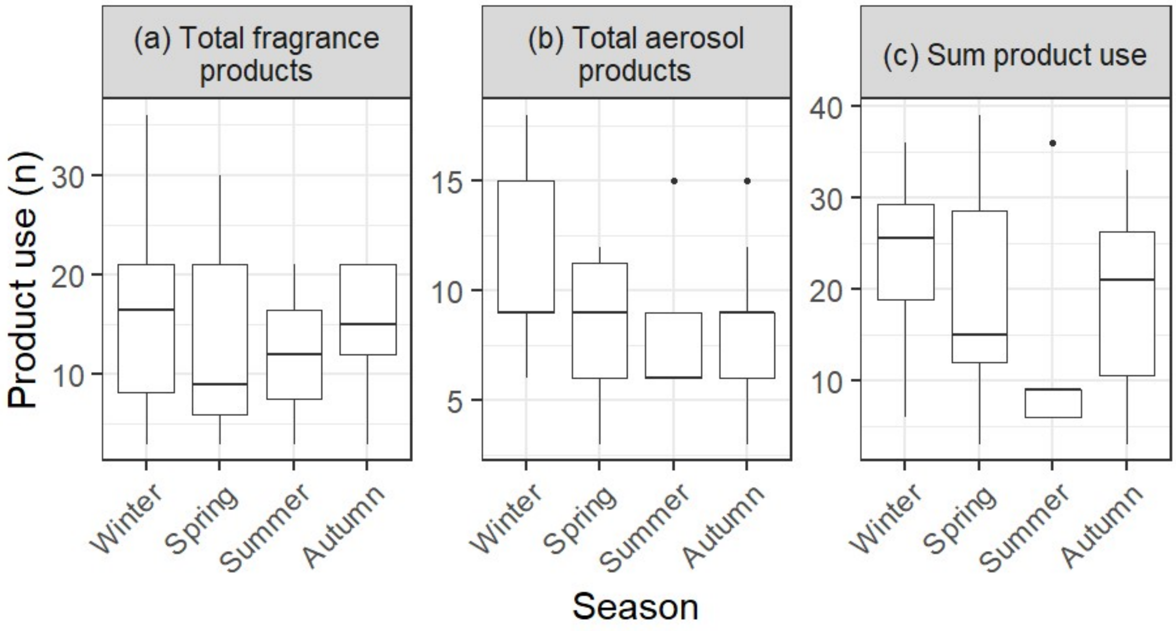

Fig. S6

Matrices of *p*-values following *post hoc* Dunn tests for seasonality in (a) summed normalised VOC emission rates and (b) summed normalised monoterpene emission rates. A white coloured matrix cell indicates the *p*-value for the pairwise comparison was not of significance. Significant values (*p*-value ≤0.05) graduate from red (*p*-value = 0.05) to blue (*p*-value -> 0)

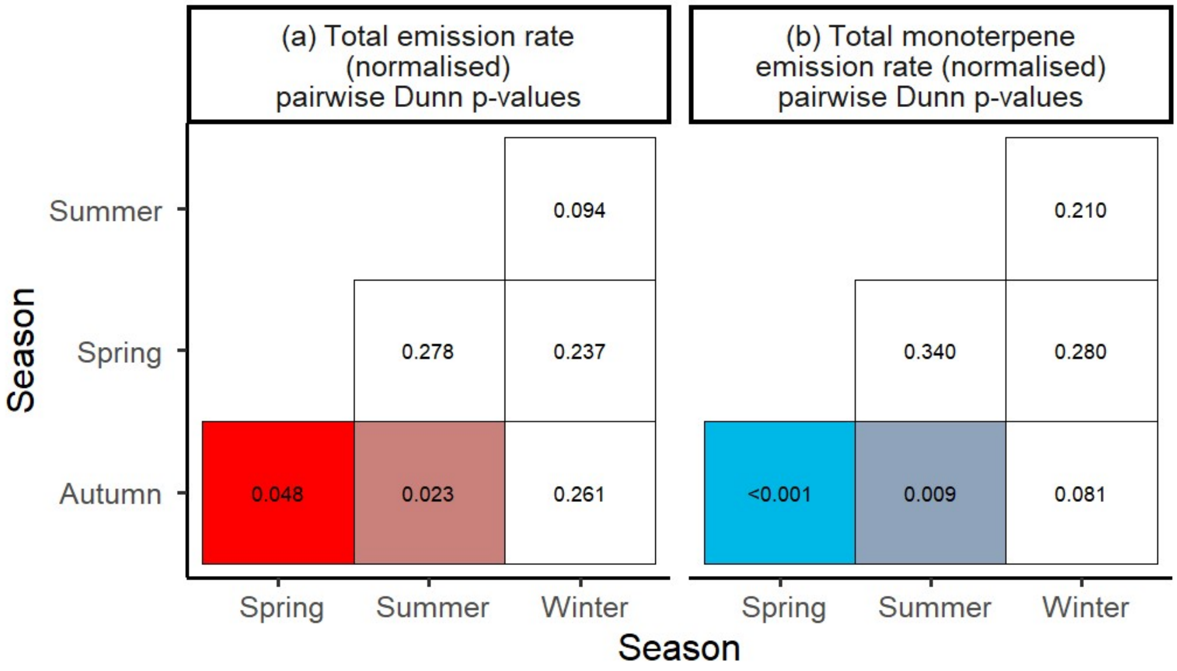

References

1. Agency, U.S.E.P. *IRIS Assessments*. 2024 [cited 2024 October 26]; Available from: https://iris.epa.gov/AtoZ/?list_type=alpha.
2. United States Environmental Protection Agency. *Basic Information about the Integrated Risk Information System* 2023 June 28, 2024]; Available from: https://www.epa.gov/iris/basic-information-about-integrated-risk-information-system.
3. Office for National Statistics. *Average hours worked and economic growth, UK: 1998 to 2022* 2024 June 28, 2024]; Available from: https://www.ons.gov.uk/economy/grossdomesticproductgdp/articles/averagehoursworkedandeconomicgrowth/2024-01-22.
4. Agency for Toxic Substances and Disease Registry. *Guidance for Inhalation Exposures*. 2021 June 28, 2024]; Available from: https://www.atsdr.cdc.gov/pha-guidance/resources/ATSDR-EDG-Inhalation-508.pdf.
